# Phenological Adaptations in *Ficus tikoua* Exhibit Convergence with Unrelated Extra-Tropical Fig Trees

**DOI:** 10.1371/journal.pone.0114344

**Published:** 2014-12-04

**Authors:** Ting-Ting Zhao, Stephen G. Compton, Yong-Jiang Yang, Rong Wang, Yan Chen

**Affiliations:** 1 Ecological Security and Protection Key laboratory of Sichuan Province, College of Life Science and Biotechnology, Mianyang Normal University, Mianyang, Sichuan 621000, China; 2 School of Biology, University of Leeds, Leeds LS2 9JT, United Kingdom; 3 Department of Zoology and Entomology, Rhodes University, Grahamstown 6140, South Africa; Saint Mary's University, United States of America

## Abstract

Flowering phenology is central to the ecology and evolution of most flowering plants. In highly-specific nursery pollination systems, such as that involving fig trees (*Ficus* species) and fig wasps (Agaonidae), any mismatch in timing has serious consequences because the plants must balance seed production with maintenance of their pollinator populations. Most fig trees are found in tropical or subtropical habitats, but the dioecious Chinese *Ficus tikoua* has a more northerly distribution. We monitored how its fruiting phenology has adapted in response to a highly seasonal environment. Male trees (where fig wasps reproduce) had one to three crops annually, whereas many seed-producing female trees produced only one fig crop. The timing of release of *Ceratosolen* fig wasps from male figs in late May and June was synchronized with the presence of receptive figs on female trees, at a time when there were few receptive figs on male trees, thereby ensuring seed set while allowing remnant pollinator populations to persist. *F. tikoua* phenology has converged with those of other (unrelated) northern *Ficus* species, but there are differences. Unlike *F. carica* in Europe, all *F. tikoua* male figs contain male flowers, and unlike *F. pumila* in China, but like *F. carica,* it is the second annual generation of adult wasps that pollinate female figs. The phenologies of all three temperate fig trees generate annual bottlenecks in the size of pollinator populations and for female *F. tikoua* also a shortage of fig wasps that results in many figs failing to be pollinated.

## Introduction

The times of year when plants flower and set seed are not random, even among plant species growing in relatively aseasonal tropical environments [1]. Flowering phenology is subject to selection from a combination of abiotic, biotic and intrinsic factors linked to life history, and also to the plant’s phylogeny [2], [3]. Abiotic factors include constraints imposed by physiological responses to temperatures, day lengths and other climatic variables, while biological factors include the availability of pollinators and seed dispersal agents and competition with other plants flowering at the same time [4], [5]. In temperate latitudes, strong climatic seasonality provides particular constraints, with threshold temperatures limiting both insect pollinator activity and the length of the period when floral and seed development can continue. This has led to widespread convergence in flowering times, most noticeably with a spring-time concentration of flowering [6], [7], despite potential competition for pollinators among animal-pollinated plants and increased likelihood of receipt of heterospecific pollen from other species flowering at the same time. Variation in flowering times also has evolutionary implications, potentially contributing to reproductive isolation and speciation [8], [9], [10].

The time of year when flowers are available to be pollinated is especially important for plants which depend on one or a small number of insect species for pollination [11]. In the case of nursery pollination systems, where the reward provided by the plant is a place for the insects to breed, any mismatch in timing has serious consequences for the population dynamics of both partners in the mutualism [12]. The more than 800 species of fig trees (*Ficus*, Moraceae) and their pollinating fig wasps (Agaonidae) are partners in a largely species-specific nursery pollination system. Fig trees are often keystone species providing food for a diverse range of fruit-eating birds, mammals and other vertebrates [13]. The significance of figs for vertebrates results from their structure, which makes them easy to eat, their abundance in a variety of habitats and what is often an all-year round fruiting phenology that makes figs available at times of the year when the fruits of other plants are absent. The all-year fruiting phenology of many *Ficus* species may be linked to their unique pollination system, because it helps maintain populations of their pollinator fig wasps, with which they have an obligate association [14]. Highly reciprocal adaptive traits and co-evolutionary dynamics are ubiquitous between fig hosts and their pollinators, and continue to stimulate ecological and evolutionary questions [15].

Adult female pollinating wasps do not feed and only survive for one or two days after emerging from their natal figs [16]. As a consequence, the synchronization of pollinator release with the production of receptive figs is critical for both the maintenance of pollinator populations and pollination of the figs [17]. Fig trees with a monoecious breeding system produce figs that support the development of both seeds and fig wasp progeny. Individual trees typically produce synchronized fig crops, each of which has a brief period when they are attractive to wasps and another when the next generation of fig wasps emerge and disperse. Tree populations contain trees that flower at different times, which ensures that the fig wasps that emerge from each tree have a chance to find suitable figs on other trees [17]. Dioecious fig tree species have female individuals with mature figs which only contain seeds and male individuals with figs that produce pollen and support the development of pollinating fig wasp offspring. The flowering phenologies of dioecious fig trees are more diverse than those of monoecious species. Figs may or may not be produced in synchronized crops on individual trees, and the timing of flowering often differs between the sexes [18], [19].

Latitudinal trends in the flowering times of plants are well documented, with selection for example tending to favour earlier flowering among plants growing at higher latitudes [20], [21]. The vast majority of *Ficus* species have tropical or sub-tropical distributions [14], a latitudinal range that appears to be related to global temperature patterns, because during a warmer period of Earth history they were also present in northern Europe [22]. A small number of fig tree species currently extend to higher latitudes [23], where they have evolved atypical fruiting phenologies in response to the strong seasonality of their environments, in particular the long winter periods that are too cold for fig wasps to be dispersing between trees.

The fruiting phenologies of three species of dioecious fig trees with largely extra-tropical distributions have been described, *F. erecta* and *F. pumila* in China including Taiwan, and *F. carica* in Europe [24], [25], [26]. All three species belong to *Ficus* subgenus *Ficus* [27] and are passively pollinated by species of *Blastophaga* and *Wiebesia* belonging to Agaonidae, Subfamily Agaoninae [25], [28]–[31]. The three species produce relatively synchronized crops, population wide, at set periods each year. The resulting precise matches between the phenologies of male and female trees facilitate the pollination of female figs while at the same time maintaining pollinator populations [25], [30]. In Europe, the pollinator of *F. carica* has larvae that overwinter in one crop of male figs. They become adults in the spring, a time when there are many receptive male figs available to lay their eggs in, often on the same plants. This allows pollinator numbers to increase, but when the next generation of pollinators emerges in the summer there are very few receptive male figs available, but many receptive female figs. Seed set is ensured, but at the expense of an annual bottleneck in pollinator populations [24], [32]. Not all individuals exhibit precisely the same phenology, and an extra generation of figs is developed in some plants [33]. In most parts of its range, *F. erecta* has a flowering phenology that is largely the same as that of *F. carica* [25]. This changes when the plant is grown under warmer conditions, outside its native range, where fig production on male trees becomes asynchronous [34]. The phenology of a second Asian species, *F. pumila,* is also similar to those of *F. carica* and *F. erecta*, but with one major difference. Like the other species, male trees produce two major crops each year, and female trees produce a single major crop. However, whereas *F. carica* and *F. erecta* build up pollinator numbers in the spring by fitting in a post-winter generation in male figs, this is not the case in *F. pumila*, where it is the generation of fig wasps that has overwintered as larvae that emerges at the same time as female figs are receptive, and so contributes to seed set. Based on the phylogenetic relationships of the plants (and also their pollinators), the three plants are not closely related species [35], [36] and their atypical phenologies represent convergent responses to selection pressures generated by the seasonality of their environments.

*F. tikoua* has been assigned to *Ficus* Subgenus *Ficus* [27], but molecular evidence [36] and also its morphology (F. Kjellberg, Pers. Comm.) show that it should be placed in *Ficus* Subgenus *Sycomorus,* which contains both monoecious and dioecious species. It is pollinated by an undescribed species of *Ceratosolen* (Agaonidae, Subfamily Kradibiinae). Both the tree and its pollinator are distantly related to the other dioecious fig trees with northern distributions. Furthermore, all described *Ceratosolen* species are active pollinators [36], in contrast to the passive pollinators of the other three species. Active pollinators collect pollen into thoracic pollen pockets before leaving their natal figs. After entry into a receptive fig the pollen is actively removed and deposited on the stigmas. This behavior is carried out even if the fig wasp has entered a female fig and is unable to oviposit there [37]. In passively pollinated fig trees the insect makes no direct effort to collect or transport pollen, and pollination is dependent on pollen grains that were transported on the body of the insect. This form of pollen transfer is less efficient, and requires male plants to produce more pollen. This relative inefficiency is reflected in the much larger number of male flowers present in figs of passively pollinated fig trees [28].

Each year, only one of the generations of fig wasps that emerges from male figs of *F. carica, F. erecta* and *F. pumila* has a high probability of entering female rather than male figs and thereby contribute to the reproductive success of the male plants from which they emerged. Pollen carried by the pollinators released at other times of the year represents a metabolic cost for which there is no direct reward to the plants. Reflecting this, figs on male trees of *F. carica* and *F. pumila* that are produced at other times of the year do not contain functional male flowers, so the pollinators that emerge from them carry no pollen [24], [38]. These species are passively pollinated, and their pollinators can clearly develop successfully in male figs that receive no pollen. It is unclear whether *F. erecta* is similar.

Unlike the other northern species, *F. tikoua* has an active pollinator, which may limit its ability to produce pollen-free male figs if pollen aids pollinator fecundity [39]. Here, we studied the fruit phenology of *F. tikoua* within its native range in China and address the following questions: (1) what is the flowering phenology of *F. tikoua* and does it vary between the sexes? (2) what proportion of the figs produced by *F. tikoua* are entered by pollinators and does this vary with season? (3) does this species exhibit convergence in reproductive phenology with *F. carica*, *F. erecta* and *F. pumila*? And if so (4) are male flowers present in male figs throughout the year, as in most fig trees, or are female-flower only figs produced seasonally, as recorded for two of the other extra-tropical dioecious fig tree species?

## Materials and Methods

### Ethics Statement

Our sampling site was not in a national park or protected area. The studies species, *Ficus tikoua*, is not an endangered or protected species, so specific permission was not required. The specific location of the sampling site is 31.45°N, 104.60°E.

### *Ficus tikoua* and its fig wasps

The natural distribution of *F. tikoua* Bureau covers Southwest and Central China and montane areas of Northeast India, Laos and North Vietnam, where it is found in wastelands, grassy banks, rocky areas and open woodland [40]. It is a prostrate shrub that does not reach a height of more than about 30 centimeters, with figs located at the leaf axils. The figs are often partially buried in the soil and for this reason it is called “di-guo” in Chinese, meaning ‘fruit from soil’. The figs are small, flattened ovoid, reaching 10–20 mm in diameter at maturity. Both male and female figs remain yellow-brown when ripe, suggesting that terrestrial mammals may contribute to seed dispersal [13].

Genetic differentiation between adjacent *F. tikoua* populations suggests that its *Ceratosolen* sp. pollinator disperses less widely than the pollinators of most *Ficus* species [41]. This may be a consequence of the plant’s small crops and the cryptic location of its figs, all of which reduce the ‘apparency’ of *F. tikoua* to pollinators, and makes long distance detection of suitable figs more difficult. The fig wasp community associated with *F. tikoua* is simpler than that of most species of fig trees. Widespread sampling throughout most of the range of the plant has detected only the pollinator (*Ceratosolen sp. indesc.*) and one non-pollinating fig wasp (NPFW), its presumed parasitoid, a species of *Philotrypesis* (Pteromalidae, Sycoryctinae) (Y. Chen et al., unpublished). As in most dioecious fig tree species, no fig wasp offspring were recorded from female figs.

### Locality and methods

Our study population was located on a small hill with sparse deciduous forest located in Mianyang, Sichuan Province, China (31.45°N, 104.60°E), which is towards the northern edge of the natural distribution of *F. tikoua*. The region has a subtropical monsoon climate with four distinct seasons. Summers are long, hot and humid, and winters are relatively short and mild, but with some snow. Mean minimum temperatures for the coldest month (January) are about 3°C, and mean maximum temperatures for the hottest months (July and August) are about 30°C (http://www.chinaweatherguide.com/sichuan/mianyang-weather.htm).

The creeping growth form of *F. tikoua* makes it difficult to distinguish between individuals and to identify which figs are produced by each individual. We therefore established sampling points with dense *F. tikoua* foliage that were at least 30 meters apart between each other that were assumed to represent 32 different plants. One meter square areas were marked at each point. The numbers and developmental stages of the figs in each square were generally recorded every seven to 10 days between 30 November 2012 and 2 March 2014, but recorded monthly during the winter periods. Fig developmental phases were assigned based on the scheme of Galil [42]. A phase figs are immature, B phase figs are the stage when pollinators enter, C phase is when fig wasp offspring and seeds develop, D phase is when fig wasp offspring vacate male figs and E phase female figs have become attractive to seed dispersers. To follow the growth of individual figs, we also marked 20 randomly-chosen figs in each square with small plastic labels. If less than 20 figs were present the nearest figs outside the squares were also marked. The maximum diameters of the labelled figs were measured with electronic Vernier calipers. When one of these marked figs aborted it was replaced by a nearby fig whenever possible.

To compare the relationship between weight and diameter of male and female figs, and to relate size to developmental stages, we also sampled 15 to 20 figs at random away from the marked areas once a month between February and December 2013. Their maximum diameters and fresh weights were measured. Late C phase male figs were also collected from outside the marked areas and kept in netting bags to let the fig wasps emerge naturally. The figs were then dissected to remove any remaining fig wasps from inside and their contents recorded.

Seasonal differences in male and female flower numbers and fig wasp contents in male figs were tested using Generalized Linear Models (GLMs) assuming quasiPoisson distribution of residuals. Pair-wise comparisons were carried out using multiple tests with Bonferroni correction. All analyses were carried out using R [43].

## Results

No sampling squares were recorded to produce both male and female figs. Among 32 sampling squares, 20 produced male and 8 produced female figs, so they were regarded as male and female plants respectively. The rest four sampling squares/plants produced no figs within our observation period.

### The phenology of *F. tikoua*

**Figure 1:**
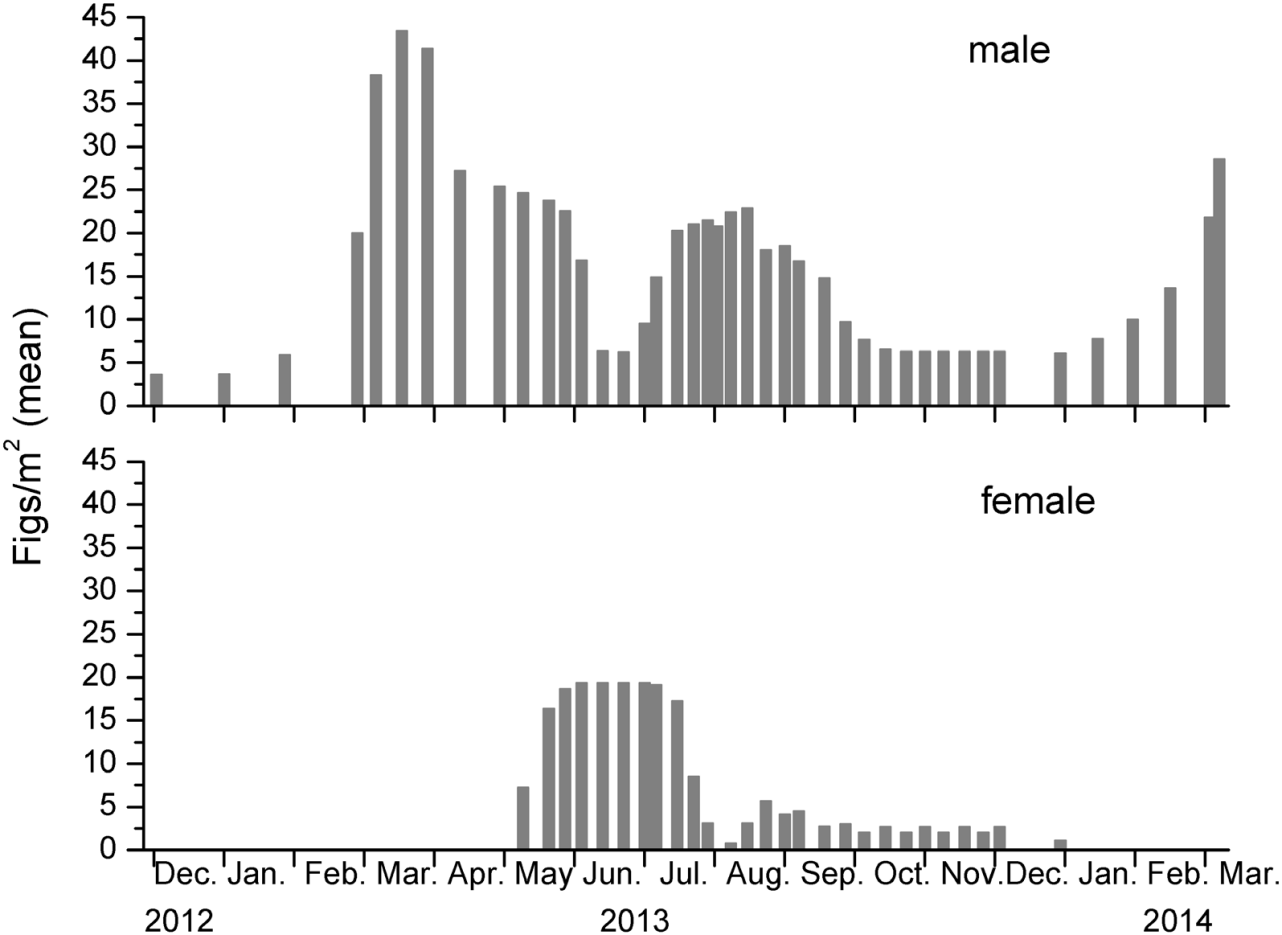
Seasonal variation in the densities of figs from 28 demarcated areas of *Ficus tikoua* plants in Mianyang.

Peak numbers of figs in the 1 m^2^ areas were noticeably higher on the male plants, with maximum densities recorded on the 20 males ranging from 26 to 223, compared with 15 to 28 figs on female plants. Figs were present on some of the male plants throughout the year, but were only at very low densities during the winter months (Figure 1, Figure S1 in File S1). A spring burst of fig production resulted in the highest average density of male figs being recorded in March–April, followed by a gradual decline through to the end of the year, interrupted by a second, smaller burst of fig production in early Autumn (Figure 1). Female figs were not recorded during the winter. They were mainly produced during the summer months, but small numbers were recorded on some plants through to the start of the winter (Figure 1).

**Figure 2:**
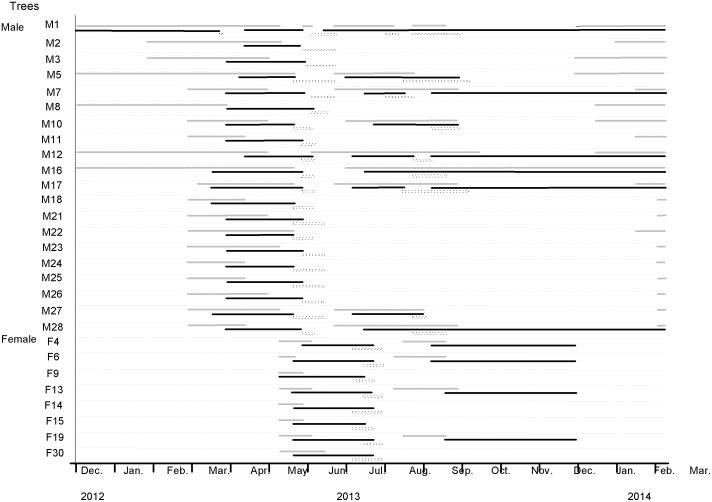
The fruiting phenologies of 20 male (M) and 8 female (F) *Ficus tikoua* in Mianyang. Phases A and B are hard to distinguish and are combined here. Grey and black bars indicate Phases AB and C, respectively. The D phase of male individuals and E phase of female individuals is shown by dotted bars.

Figs were also absent from most of the demarcated areas on male plants during the winter months, and pollinated figs (that contained fig wasp offspring) were recorded from just one of the plants during the first winter (Figure 2). In the following spring, all 20 male plants had young (phase AB) figs present and the first of these were entered by fig wasps (phase C) in late March and early April. Just one fig from within the 20 demarcated squares released fig wasps at that time, so most of the pollinators that entered the B phase figs in spring will have emerged from figs outside our sampling squares. The next generation of adult pollinators emerged in early summer (late May and June), when very few AB phase figs were present on the male plants (Figure 2). Fig production resumed on some of the male plants in mid-late summer, and these supported a generation of pollinators that emerged in autumn (Figure 2). Immature figs were present in a few of the sampling areas at that time, providing oviposition sites for the autumn fig wasps and allowing the fig wasp population to persist on five of the plants through the second winter. In summary, across the sampling period as a whole, the demarcated areas of all the male plants released pollinators in early summer and about half also released smaller numbers of pollinators once or twice more later in the year (Figure 2), some of which had the offspring that overwintered.

**Figure 3:**
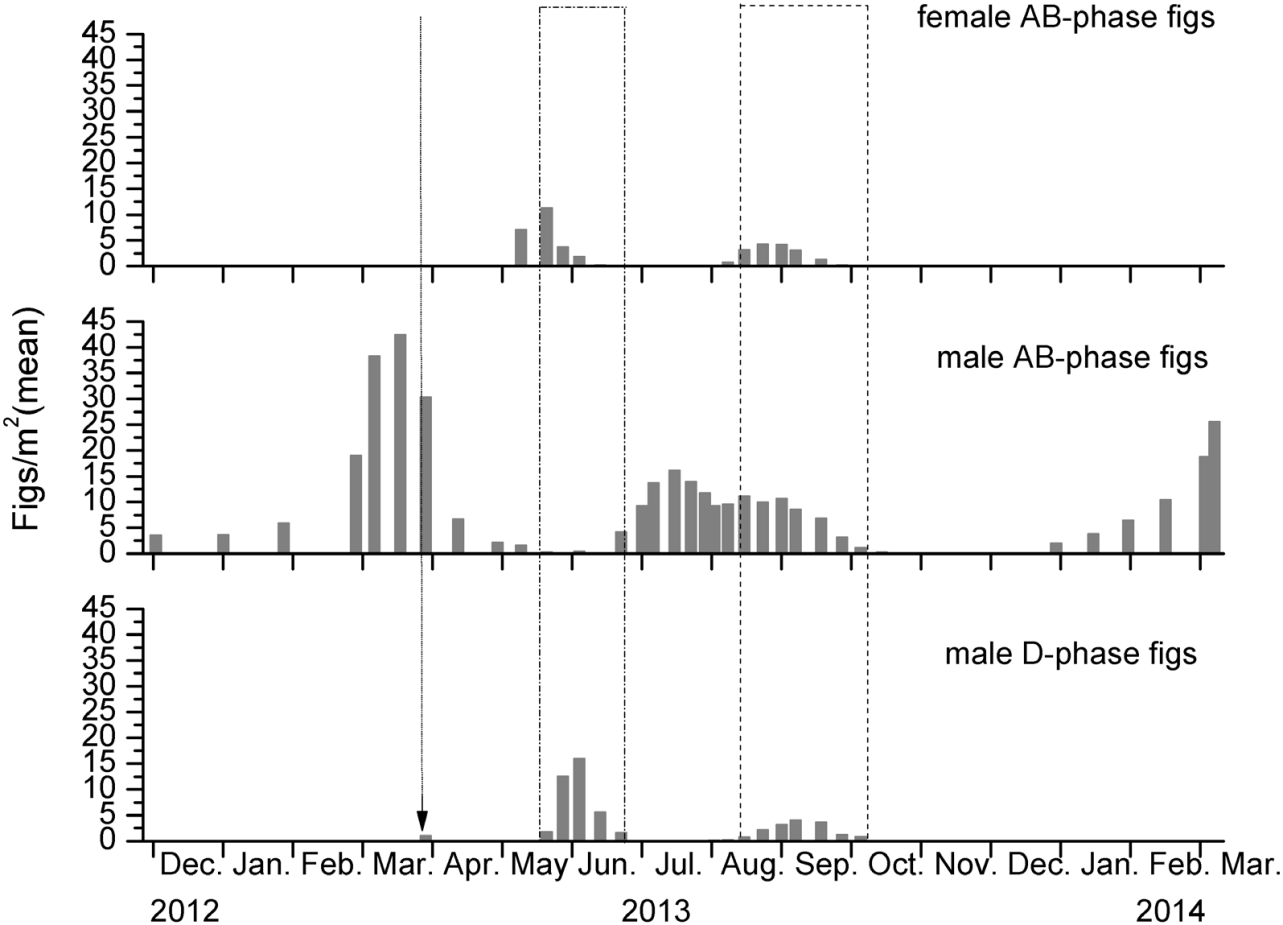
Seasonal variation of the densities of figs at different developmental stages in demarcated areas of *Ficus tikoua* plants in Mianyang. The times when pollinators were emerging (D-phase figs) of spring and early-summer crops of male plants are highlighted by dash-dot and dash frames respectively. The arrow indicates an additional isolated occurrence of D-phase male figs.

Fig production by female *F. tikoua* was concentrated in the summer months, when all eight demarcated squares contained figs (Figure 2). Several of the plants also produced a second, but much smaller, crop in the autumn, but none of these figs survived the winter (Figure 2, Table S1 in File S1). The receptive (phase B) periods of both crops of female figs corresponded closely with the periods when fig wasps were being released from male figs (phase D figs, Figures 2 & 3). The much larger early summer female crop also corresponded with the larger numbers of male figs releasing wasps at that time, and also will have benefitted from a virtual absence of competition for pollinators from B phase male figs (Figure 3). This contrasts with the smaller supplementary second crops of female figs, which was produced at a time when many of the pollinators emerging from male figs had the opportunity to enter receptive figs on the same plants.

### The development, pollination and abortion of male and female figs

Relationships between fig size and weight were recorded for 201 male and 100 female figs from outside the demarcated areas. A power-function relationship was present between their fresh weights and diameters (R^2^ = 0.91 and 0.99 for male and female figs respectively, Figure S2 in File S1). The female figs were a little heavier than male figs of similar diameters, especially after they had been pollinated (from the beginning of C-phase). However, no significant differences were found between the regression equations of each sex (likelihood ratio test, χ^2^ = 0.298, p = 0.585). The smallest female figs that had been entered by pollinators had a diameter of 6.09 mm, compared with 7.11 mm for male figs (Figure S2 in File S1), but mature female figs (E phase) were considerably larger than male figs at the time that they released pollinators (D phase) (Female figs have no equivalent to D phase and pass directly from C to E phase).

**Figure 4:**
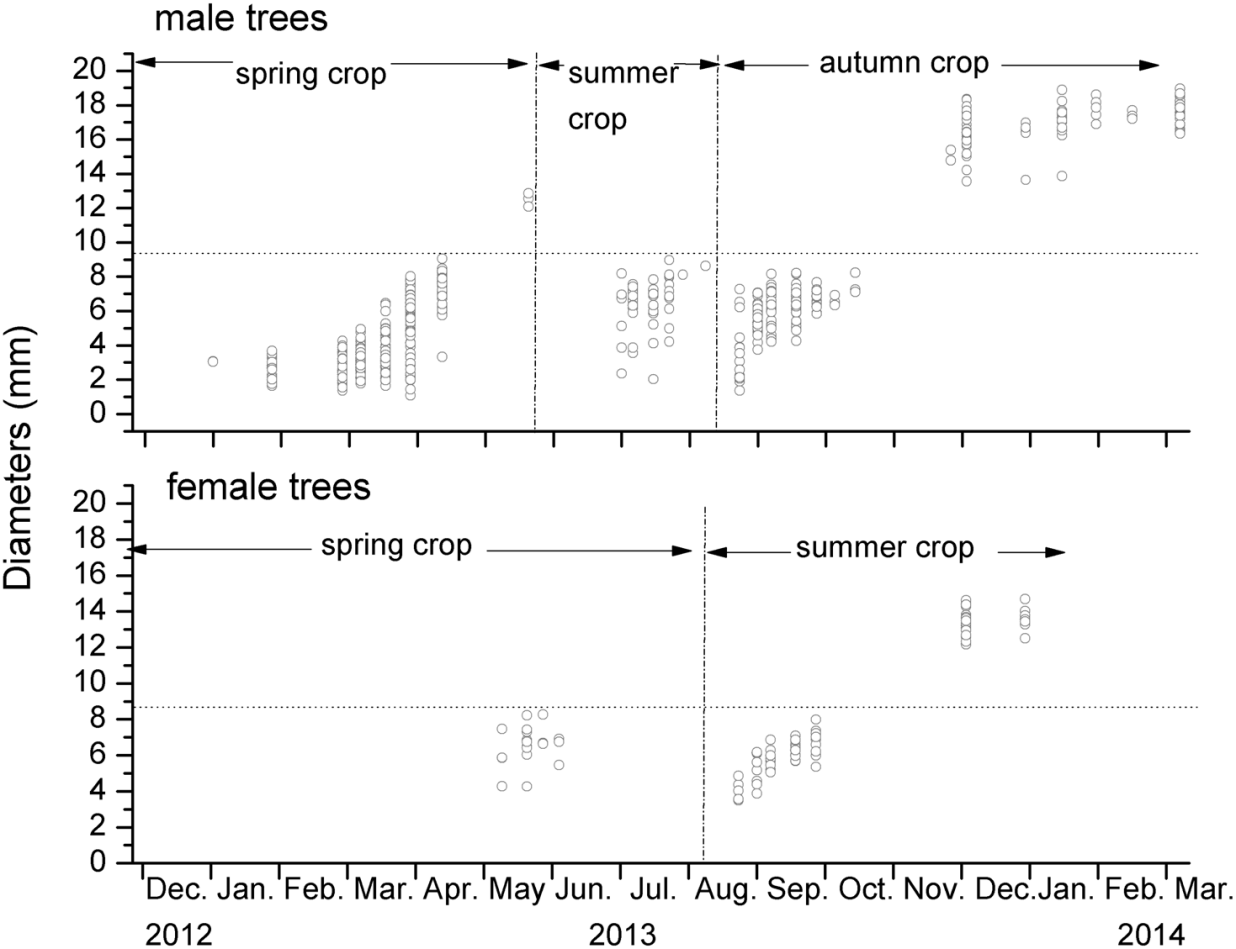
The last recorded diameters of figs of *Ficus tikoua* that became detached from the plants before they had reached maturity. The observed maximum diameter of un-pollinated figs is indicated by the horizontal dotted line. Figs which became detached before reaching these diameters had aborted after failing to attract pollinators.

Few figs could be recovered after they became detached from the plant, but repeated measurements of the same figs allowed us to decide which figs had aborted without being pollinated, based on their diameters on the last occasion when they were still attached to the plants. Figs with smaller diameters than the observed maximum diameter of un-pollinated figs were assumed to have aborted without being entered by fig wasps. Abortions were frequent among both male and female figs and ‘replacement’ figs often themselves had to be replaced after they also aborted (Table S1, S2). Overall, 62% of the initially-marked male figs (436/700) and 33% of the female figs (67/205) aborted before they matured (reached D phase if male figs, or E phase if female figs). Most of these abortions (including those from some replaced figs) took place before the figs reached the size when pollinators entered (Figure 4) and an estimated 86% (589/683) of the aborted male figs and 81% (162/200) of the aborted female figs had fallen from the plants without being entered by pollinators.

Abortion rates varied considerably between crops. Just 32% (126/400) of the initially-marked male figs in the spring crops completed their development and released fig wasp offspring, compared with 72% (130/180) of the summer crop and only 6.7% (8/120) of the autumn crop (Table S1 in File S1). Substantially different abortion rates were also found among female crops, with most spring crop figs reaching maturity (86%, 138/160), whereas no summer crop figs reached maturity (Table S2 in File S1). There were also large between-crop differences in the sizes of the figs when they aborted. Most spring and summer crop abortions among male figs occurred when the figs were small, indicating a shortage of pollinators, whereas the high abortion rates among autumn male crop figs resulted from a combination of early abortions among un-entered small figs and losses of larger, pollinated figs, through the winter period (Figure 4). Female figs showed a similar pattern (Figure 4).

### Contents of the figs

Mature figs from outside the marked areas were collected and dissected in 2013. Spring (main) crop female figs, collected in August, contained 782.00±111.55 seeds (mean ± SD, n = 30 figs). No summer crop female figs could be found. Male figs could support far fewer pollinator offspring because they contained a much smaller number of female flowers. Male flowers made up only a small proportion of the total flowers, reflecting the active pollination exhibited by *Ceratosolen* species. Pollinator sex ratios were strongly female-biased. The putative parasitoid *Philotrypesis* was always rare (Table 1).

**Table 1:** The contents of mature male figs of *Ficus tikoua* collected in Mianyang from outside the marked areas.

Crops	SampleDate	N figsFlowers/Wasps	Flowers per fig (mean ± SD)			Fig Wasps per fig(mean ± SD)		NPFW %(mean ± SD)	Sex ratio ofpollinators(mean ± SD)
			Male	Female	Total	Pollinators	Total wasps		
Autumn2012	March2013	42/0	24.31±6.11	260.44±65.89	284.74±68.60	/	/	/	/
Spring2013	June2013	80/80	25.26±5.15	209.39±44.20	234.65±45.68	186.43±40.91	205.80±44.38	0.095±0.028	0.063±0.029
Summer2013	September2013	39/24	22.90±6.62	233.33±72.93	256.23±72.22	174.88±49.36	192.58±54.40	0.092±0.020	0.098±0.040
Total		161/104	25.06±5.67	218.40±63.45	243.46±63.80	183.76±43.03	202.75±46.93	0.094±0.026	0.071±0.035

The only NPFW recorded was a species of *Philotrypesis*.

Only flower numbers were recorded from the overwintered fig crop initiated in autumn 2012 that matured in spring 2013 because the wasps had already emerged when the figs were sampled at the end of March. Numbers of male flowers in male figs did not vary among seasons (GLM: LR = 6.10, df = 2, p = 0.110), but female flower numbers were significantly higher in autumn than spring with intermediate values in summer figs (GLM: LR = 301.81, df = 2, p<0.01; pair-wise comparisons: autumn vs. spring: t = 4.57, p<0.001; autumn vs. summer: t = 2.00, p = 0.140; spring vs. summer: t = −2.189, p = 0.0903) (Figure S3 in File S1).

## Discussion

Like other dioecious fig trees, seed production in *F. tikoua* depends on pollen-carrying adult female fig wasps being available at the times when receptive figs are present on female trees. Over longer time periods, the stability of the mutualism also depends on male plants being able to support populations of their specific fig wasp pollinators. Genetic evidence [41] suggests that *F. tikoua*’s *Ceratosolen* pollinators are relatively sedentary and do not fly the long distances seen in some congeneric species [44], so populations of *F. tikoua* cannot rely on the services of fig wasps that developed elsewhere, and must maintain their own local pollinator populations. Fig wasps develop only in the figs of male plants, so it is only their flowering phenology that is constrained by the need to support the development of the pollinators, whereas the phenology of female plants is subject to the same selection pressures, from the environment and mutualists, that influence flowering times among plants in general. In Mianyang, *F. tikoua* male trees produced one to three crops annually (similar intraspecific variation in the number of fig generations has also been reported in European populations of *F. carica* [33]). This ensured that some male figs containing pollinator offspring were present locally throughout the year, though whether or not the overwintering stages entered a true diapause is unclear. The fruiting phenology of *F. tikoua* results in pollinator populations going through two bottlenecks each year, once during the winter and another when many of the adult pollinators become trapped in female figs. These bottlenecks are temporary, because male trees produced additional crops with sufficient figs to allow subsequent generations of pollinators to recover. Female plants produced a single annual major crop of figs that was pollinated in late spring. Pollination was facilitated by synchrony with the release of pollinators from the main crop of male trees that bear few receptive figs at that time. Smaller numbers of receptive figs were present on female trees later in the year, but at that time they were competing with male trees to attract pollinators and it was not confirmed whether any of this later crop set seed successfully. Figs on male plants contain male flowers throughout the year, even though it is only those figs that release fig wasps in late spring that are likely to contribute to their reproductive success. At other times, pollen carried into male figs can nonetheless indirectly benefit pollen donor trees if it increases the fecundity of pollinators, but only if these fig wasps have entered receptive figs on the same plant [45]. If they enter figs on other male plants then there is no benefit accruing from pollen production.

The evolution of a dioecious breeding system provided many potential advantages for fig trees relative to their ancestral monoecious breeding system, including avoidance of self-pollination, the potential to chemically defend ovules from non-pollinating fig wasps and the partial decoupling of flowering times of male and female individuals [46], [47]. Tropical dioecious fig trees exhibit a wide range of phenologies, including seasonal concentrations of flowering that allow peaks in pollinator release from male plants to coincide with peaks in the numbers of female figs waiting to be pollinated [14], [23], [48], [49]. The flowering phenologies of extra-tropical dioecious species can be seen as extensions of these to cope with a cold winter period.

The flowering phenology of *F. tikoua* in Sichuan Province, China shows striking convergence with that of *F. pumila* and *F. erecta* elsewhere in China and *F. carica* in Europe, despite being unrelated to these other temperate dioecious fig trees. The independently-evolved similarities in fruiting patterns that they exhibit illustrate the likely constraints acting on fig trees growing under strongly seasonal conditions, combined with vicariant selection operating reciprocally on the two sexes of the plants to flower at appropriate times [39]. Low temperatures limit the rate of pollinator larval development, when adult male fig wasps can chew exit holes to allow emergence of their females and the times of year when females can disperse and carry pollen between male and female fig trees. Temperatures also influence rates of seed development and germination, and the ability of seedlings to establish. The timing of mature seed production in *F. tikoua* may simply be as early in the year as can be achieved given pollination constraints, but their fruiting phenology has a clear benefit in that it avoids the need for figs containing developing seeds to be retained on female plants through the winter period.

*F. tikoua* is the only one of the four temperate dioecious fig trees to benefit from active pollination of its flowers. Inflorescence structure in figs from different crops of male *F. erecta* has not been compared, but in *F. carica* and *F. pumila* only one crop of male figs each year contains male flowers - the crop that is synchronized with the availability of female figs to pollinate. Pollen production is more costly for these passively-pollinated species than in the actively-pollinated *F. tikoua*, because they need to produce more pollen to achieve adequate fertilization. Passive pollinators haphazardly distribute pollen within the male and female figs they enter, but fertilization of female flowers in male figs is inhibited [50]. Because *F. tikoua* is actively pollinated, its male figs contain fewer male flowers than would be required for passive pollination [28], so the cost of retaining male flowers is lower and selection for their loss in figs produced at times of year when no female figs are available is likely to be less than in passively-pollinated species. Furthermore, if some second crop female figs do manage to survive to maturity, even at very low frequencies, then benefits would accrue to male plants that are releasing pollen-carrying pollinators in late summer.

Results from other fig tree species where pollination is active provide an alternative or additional explanation for the retention of male flowers in all crops of *F. tikoua.* The behavior of adult female fig wasps that actively pollinate fig flowers increases the likelihood that their larvae will develop in pollinated flowers [50]. Experiments where pollinator foundresses that lack pollen are introduced into figs suggest that actively-pollinating species often suffer reduced reproductive success, due to increased larval mortalities, whereas passively pollinating species appear not to benefit from pollination [51], [52]. The presence of male flowers in male figs of *F. tikoua* throughout the year may therefore also reflect selection on the trees acting via pollinator fecundity.

Reproduction by fig trees is often limited by the number of figs entered by fig wasps [53], [54], but abortion rates in the autumn and spring crops of male and late summer crop of female *F. tikoua* seem particularly high. This resulted from a combination of a lack of pollinators and over-wintering losses among autumn-crop figs and a shortage of pollinators that had survived the winter and became available to pollinate the spring male crop. In contrast, abortion rates among the main late-spring crops of female figs were much lower, and emphasize that the plant’s phenology delivers effective pollination and seed production despite the seasonal lows in pollinator populations that it generates.

*F. tikoua* is a short creeping plant with rather small crops of small, inconspicuous figs. As such, the plant and its figs have a low ‘apparency’ to insects and they are likely to be hard to find from long distances [55], [56]. Perhaps reflecting this, its pollinators rarely disperse far [41]. Our study was carried out in an area with a large, dense population of *F. tikoua* that was clearly able to maintain a resident population of pollinators. Small founder populations may not be able to do so, and vegetative reproduction may prove to be a significant component of the plant’s overall reproductive strategy.

## Supporting Information

File S1 Figure S1. Changes in densities of phase AB figs over time in marked areas on six male *Ficus tikoua* individuals in Mianyang that produced three crops during the sampling period. Figure S2. The relationship between fresh weights and diameters of male and female figs of *Ficus tikoua* in Mianyang. Figure S3. The numbers of flowers in male *Ficus tikoua* figs from Mianyang. Table S1. Abortion rates of figs from one metre square sections of twenty male *Ficus tikoua* individuals in Mianyang. Table S2. Abortion rates of figs from one metre square sections of eight female Ficus tikoua individuals in Mianyang.
(DOCX)
