# Supplementary material for: Phenological Adaptations in Ficus tikoua Exhibit Convergence with Unrelated Extra-Tropical Fig Trees
Source: PLoS One. 2014 Dec 4;9(12):e114344. doi: 10.1371/journal.pone.0114344 (PMC4256256; doi:10.1371/journal.pone.0114344)

Table S1. Abortion rates of figs from one metre square sections of twenty male *Ficus tikoua* individuals in Mianyang. Twenty figs were marked for each individual. The dropped figs were replaced whenever possible, but the replaced figs are not included in the calculations of abortion rates. Abortion rates were calculated based only on the initially marked figs. Non-replaced means the marked figs stayed there from the first time we marked for each season to maturity.

| Tree  ID | Spring Crops | |  | Summer Crops | |  | Autumn Crops | |
| --- | --- | --- | --- | --- | --- | --- | --- | --- |
|  | non-replaced figs | abortion rates  (%) |  | non-replaced figs | abortion rate  (%) |  | non-replaced figs | abortion rates  (%) |
| M1 | 0 | 100 |  | 15 | 25 |  | 3 | 85 |
| M2 | 1 | 95 |  | / | / |  | / | / |
| M3 | 7 | 65 |  | / | / |  | / | / |
| M5 | 3 | 85 |  | 14 | 30 |  | / | / |
| M7 | 4 | 80 |  | 19 | 5 |  | 3 | 85 |
| M8 | 10 | 50 |  | / | / |  | / | / |
| M10 | 5 | 75 |  | 17 | 15 |  | / | / |
| M11 | 4 | 80 |  | / | / |  | / | / |
| M12 | 8 | 60 |  | 9 | 55 |  | 1 | 95 |
| M16 | 9 | 55 |  | 16 | 20 |  | 0 | 100 |
| M17 | 6 | 70 |  | 11 | 45 |  | 0 | 100 |
| M18 | 4 | 80 |  | / | / |  | / | / |
| M21 | 2 | 90 |  | / | / |  | / | / |
| M22 | 11 | 45 |  | / | / |  | / | / |
| M23 | 7 | 65 |  | / | / |  | / | / |
| M24 | 7 | 65 |  | / | / |  | / | / |
| M25 | 12 | 40 |  | / | / |  | / | / |
| M26 | 6 | 70 |  | / | / |  | / | / |
| M27 | 15 | 25 |  | 18 | 10 |  | / | / |
| M28 | 5 | 75 |  | 11 | 45 |  | 1 | 95 |
| Total | 126 | / |  | 130 | / |  | 8 | 93 |
| Mean | 6.3 | 69 |  | 14.4 | 28 |  | 1.3 | 93 |

Table S2. Abortion rates of figs from one metre square sections of eight female *Ficus tikoua* individuals in Mianyang. Twenty figs were marked in each squares whenever possible. The dropped figs were replaced whenever possible, but the replaced figs are not included in the calculations of abortion rates. Abortion rates were calculated based only on the initially marked figs. Non-replaced means the marked figs stayed there from the first time we marked for each season to maturity.

| Tree ID | Late Spring Crop | | |  | Late Summer Crop | | |
| --- | --- | --- | --- | --- | --- | --- | --- |
|  | marked figs | non-replaced figs | abortion rates  (%) |  | marked figs | non-replaced figs | abortion rates  (%) |
| F4 | 20 | 20 | 0 |  | 12 | 0 | 100 |
| F6 | 20 | 19 | 5 |  | 16 | 0 | 100 |
| F9 | 20 | 20 | 0 |  | - | - | - |
| F13 | 20 | 17 | 15 |  | 9 | 0 | 100 |
| F14 | 20 | 15 | 25 |  | - | - | - |
| F15 | 20 | 18 | 10 |  | - | - | - |
| F19 | 20 | 13 | 35 |  | 8 | 0 | 100 |
| F30 | 20 | 16 | 20 |  | - | - | - |
| Total | 160 | 138 | - |  | 45 | 0 | - |
| Mean | 20 | 17.3 | 13 |  | 11.3 | 0 | 100 |

Figure S1. Changes in densities of phase AB figs over time in marked areas on six male *Ficus tikoua* individuals in Mianyang that produced three crops during the sampling period. The tree identity numbers (M1 etc.) are indicated.


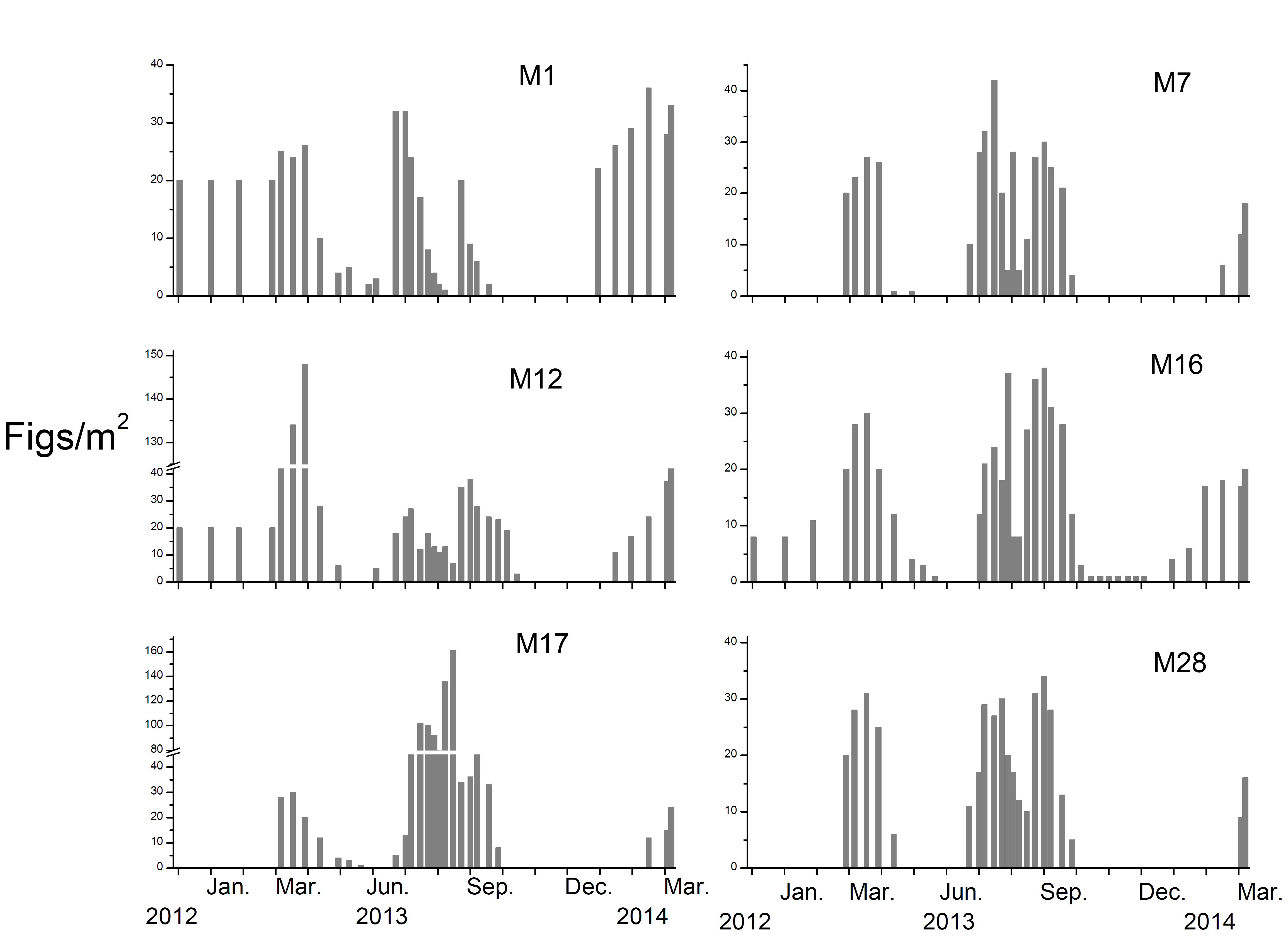


Figure S2. The relationship between fresh weights and diameters of male (open circles, N=201) and female (grey triangles, N=100) figs of *Ficus tikoua*. The power function trend lines of female and male figs are indicated by grey solid and black dashed lines respectively. The observed minimum diameters of early C-phase, male D-phase and female E phase figs are highlighted by arrows.


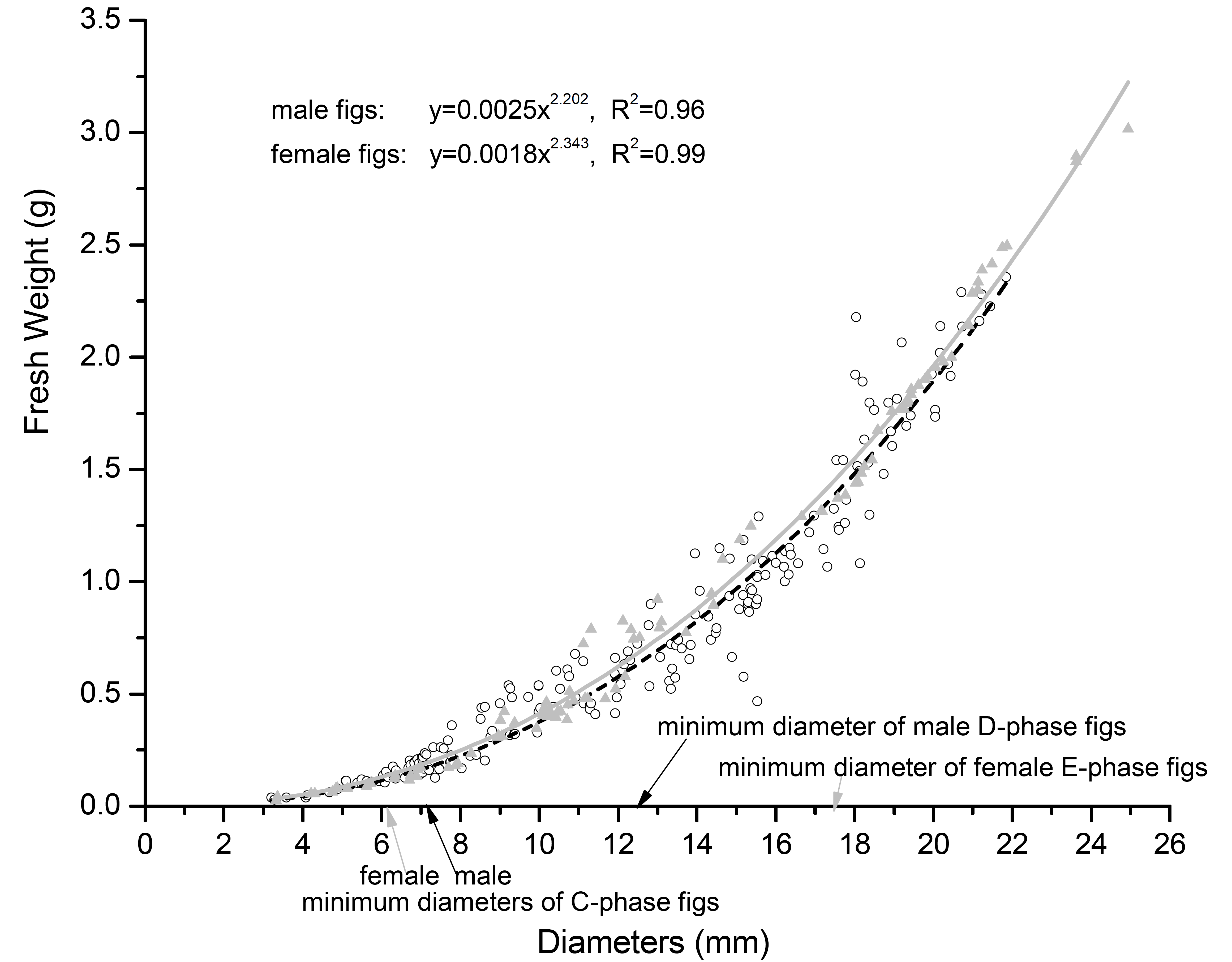


Figure S3. The numbers of flowers in male *Ficus tikoua* figs from Mianyang (open circles male flowers, black squares female flowers).


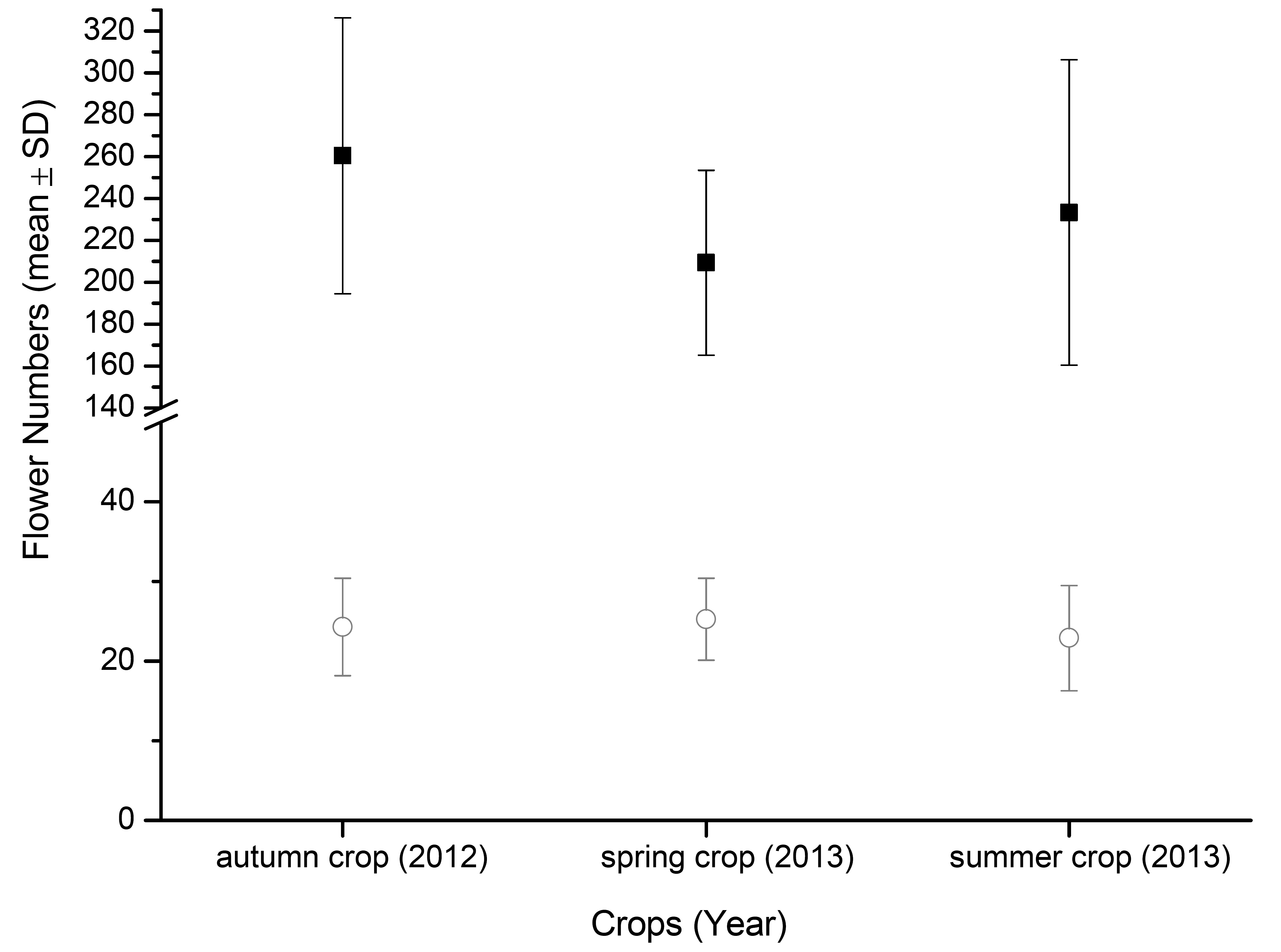

Supplement: File S1 — Figure S1. Changes in densities of phase AB figs over time in marked areas on six male Ficus tikoua individuals in Mianyang that produced three crops during the sampling period. Figure S2. The relationship between fresh weights and diameters of male and female figs of Ficus tikoua in Mianyang. Figure S3. The numbers of flowers in male Ficus tikoua figs from Mianyang. Table S1. Abortion rates of figs from one metre square sections of twenty male Ficus tikoua individuals in Mianyang. Table S2. Abortion rates of figs from one metre square sections of eight female Ficus tikoua individuals in Mianyang. (DOCX) [file pone.0114344.s001.docx]
